# Supplementary material for: The Impact of Human Milk Oligosaccharides on Antibiotic-Induced Microbial Dysbiosis and Gut Inflammation in Mice
Source: Antibiotics (Basel). 2025 May 10;14(5):488. doi: 10.3390/antibiotics14050488 (PMC12108310; doi:10.3390/antibiotics14050488)
Supplement: Supplementary file 1 [file antibiotics-14-00488-s001.zip › Table S2 Gene expressions.docx]

## Table S2

Ileal gene expressions (log2 relative quantities) in 48 female BALB/cJBomTac mice divided into six groups of eight mice with four mice in each of two cages supplied in their drinking (tap) water with HMO’s either as 2’FL alone or 2’FL and DFL in combination, either with or without ampicillin for three weeks from the age of four weeks. p and q values were considered significant if p/q < 0.05 (bold) and borderline if p/q < 0.10 (italics).

|  | **No ampicillin** | | | | | | | | | **Ampicillin** | | | | | | | | |
| --- | --- | --- | --- | --- | --- | --- | --- | --- | --- | --- | --- | --- | --- | --- | --- | --- | --- | --- |
|  | Control | | | 2'FL | | | 2'FL/DFL | | | Ampicilin | | | +2´FL | | | +2´FL/DFL | | |
| *Arg1* | 2.99144 | ± | 0.921125 | 3.38774 | ± | 1.28563 | 2.81697 | ± | 1.31955 | 3.22733 | ± | 0.381222 | 3.45102 | ± | 1.20269 | 3.57278 | ± | 0.914457 |
| *Ccl2* | 3.75539 | ± | 1.17266 | 4.38721 | ± | 0.615619 | 4.54165 | ± | 0.820219 | 3.08154 | ± | 1.3466 | 4.45694 | ± | 3.05418 | 3.3467 | ± | 1.99617 |
| *Ccl3_1* | 7.89441 | ± | 0.988144 | 7.61266 | ± | 1.01087 | 7.79534 | ± | 1.18527 | 5.11097 | ± | 2.83107 | 7.67718 | ± | 1.154 | 7.16863 | ± | 0.655257 |
| *Ccr10* | 3.20555 | ± | 1.42508 | 3.57546 | ± | 1.48013 | 3.61002 | ± | 0.932245 | 1.8619 | ± | 1.30744 | 3.17011 | ± | 0.974708 | 3.21123 | ± | 2.14674 |
| *Ccr9* | 2.9538 | ± | 0.825299 | 3.36388 | ± | 0.488586 | 2.80174 | ± | 1.01656 | 1.87627 | ± | 1.14247 | 3.68222 | ± | 1.67263 | 2.2647 | ± | 1.61242 |
| *Cd38* | 1.86242 | ± | 0.643037 | 2.40032 | ± | 0.846451 | 2.18874 | ± | 0.99118 | 2.16127 | ± | 1.32332 | 1.63428 | ± | 1.01124 | 1.87394 | ± | 1.09583 |
| *Cd3e* | 3.55922 | ± | 1.03892 | 3.34691 | ± | 1.14555 | 3.66632 | ± | 0.82839 | 1.8624 | ± | 0.909822 | 3.21638 | ± | 2.72426 | 2.33089 | ± | 1.61117 |
| *Cd8a* | 2.95112 | ± | 1.02706 | 3.0073 | ± | 1.06296 | 3.27291 | ± | 0.849099 | 1.34045 | ± | 1.16596 | 2.5553 | ± | 2.06566 | 2.26936 | ± | 1.48485 |
| *Cdh1* | 0.946764 | ± | 0.300812 | 0.859009 | ± | 0.182414 | 0.654382 | ± | 0.319451 | 0.767331 | ± | 0.36398 | 0.83631 | ± | 0.384637 | 1.08518 | ± | 0.335829 |
| *Cgn* | 1.19663 | ± | 0.208303 | 1.01664 | ± | 0.240244 | 1.05603 | ± | 0.282598 | 1.04985 | ± | 0.358828 | 0.951802 | ± | 0.495799 | 0.910854 | ± | 0.523106 |
| *Cldn1* | 4.42602 | ± | 1.28175 | 3.3788 | ± | 1.79678 | 4.03223 | ± | 0.751468 | 3.6387 | ± | 1.40914 | 4.82586 | ± | 2.07843 | 4.64828 | ± | 1.74052 |
| *Csf2* | 2.09405 | ± | 1.32674 | 2.56033 | ± | 1.48597 | 2.92743 | ± | 1.32905 | 1.85381 | ± | 0.840641 | 2.49161 | ± | 0.688041 | 1.92088 | ± | 1.50883 |
| *Ctla4* | 3.00822 | ± | 2.2424 | 3.76712 | ± | 0.93091 | 4.61788 | ± | 0.661821 | 2.71363 | ± | 1.50721 | 4.32607 | ± | 3.31179 | 1.76736 | ± | 2.30737 |
| *Ctnnb1* | 1.30929 | ± | 0.185756 | 1.38903 | ± | 0.224841 | 1.15417 | ± | 0.577555 | 1.2083 | ± | 0.210298 | 1.45091 | ± | 0.417651 | 1.4115 | ± | 0.170993 |
| *Cxcl1* | 4.06847 | ± | 0.587909 | 3.68258 | ± | 0.706428 | 3.59591 | ± | 0.91291 | 3.09537 | ± | 0.722402 | 2.87561 | ± | 2.58291 | 2.74201 | ± | 1.99536 |
| *Cxcl16* | 1.25965 | ± | 0.653484 | 1.26482 | ± | 0.501665 | 1.528 | ± | 0.506287 | 0.825689 | ± | 0.587838 | 1.76798 | ± | 0.837733 | 1.2963 | ± | 0.532638 |
| *Cxcl19* | 3.84247 | ± | 1.1197 | 3.56853 | ± | 0.993614 | 4.12947 | ± | 0.756991 | 2.60418 | ± | 1.37994 | 3.0323 | ± | 2.33451 | 2.74098 | ± | 1.78854 |
| *Cxcr6* | 2.61579 | ± | 0.542234 | 2.90623 | ± | 0.682447 | 3.04746 | ± | 0.595612 | 1.38734 | ± | 0.839158 | 2.86889 | ± | 1.33729 | 1.87169 | ± | 1.35389 |
| *Defa3* | 1.54887 | ± | 0.498396 | 1.50168 | ± | 0.362012 | 1.44033 | ± | 1.03936 | 2.08161 | ± | 0.434849 | 1.14495 | ± | 0.61351 | 2.11627 | ± | 0.630411 |
| *Defa5* | 2.49147 | ± | 0.188224 | 2.53058 | ± | 0.296291 | 2.14011 | ± | 0.868792 | 2.45306 | ± | 1.16448 | 1.98175 | ± | 0.535631 | 2.72541 | ± | 0.55016 |
| *Epx* | 2.64894 | ± | 1.25686 | 2.33996 | ± | 0.91217 | 1.55537 | ± | 0.801904 | 2.62568 | ± | 1.15464 | 2.78221 | ± | 0.665746 | 2.39782 | ± | 2.33708 |
| *Fasl* | 5.48883 | ± | 0.761733 | 5.06617 | ± | 0.701574 | 4.95986 | ± | 2.24493 | 4.76159 | ± | 0.812996 | 5.40614 | ± | 1.54547 | 4.28189 | ± | 1.60004 |
| *Ffar4* | 3.44441 | ± | 0.405095 | 3.62256 | ± | 0.604783 | 3.25847 | ± | 1.52044 | 3.7005 | ± | 0.431327 | 4.26351 | ± | 0.444701 | 3.20464 | ± | 0.34162 |
| *Fgf15* | 4.9895 | ± | 1.1797 | 4.53945 | ± | 1.75377 | 3.75981 | ± | 1.89591 | 4.32545 | ± | 1.85051 | 2.89442 | ± | 1.79368 | 4.25541 | ± | 2.324 |
| *Foxp3* | 1.66882 | ± | 1.10486 | 1.64024 | ± | 0.914985 | 1.91244 | ± | 1.003 | 1.82805 | ± | 0.790657 | 3.03583 | ± | 2.93929 | 1.80223 | ± | 1.15984 |
| *Fxr* | 0.906114 | ± | 0.23009 | 0.9434 | ± | 0.428915 | 0.807801 | ± | 0.448316 | 0.849322 | ± | 0.592111 | 0.809808 | ± | 0.578312 | 1.09934 | ± | 0.436863 |
| *Gata3* | 2.95807 | ± | 1.03966 | 2.71097 | ± | 1.28826 | 3.62384 | ± | 0.884607 | 1.81898 | ± | 0.53811 | 3.73123 | ± | 2.51362 | 2.00361 | ± | 0.89661 |
| *Gcg* | 3.21407 | ± | 0.268025 | 3.10216 | ± | 0.450468 | 2.44747 | ± | 1.18176 | 3.22756 | ± | 0.281949 | 3.10278 | ± | 0.469729 | 3.36046 | ± | 0.681437 |
| *Gzmb* | 4.34476 | ± | 1.23515 | 4.6243 | ± | 1.37146 | 5.21946 | ± | 0.73935 | 2.56015 | ± | 1.75875 | 2.85841 | ± | 3.09398 | 3.17114 | ± | 1.49197 |
| *Icam1* | 3.13885 | ± | 1.29328 | 3.04841 | ± | 0.985351 | 3.23502 | ± | 0.60072 | 1.82113 | ± | 1.00409 | 2.85496 | ± | 2.20532 | 1.74476 | ± | 1.09456 |
| *Il10* | 3.20158 | ± | 1.77899 | 3.79968 | ± | 1.36382 | 4.142 | ± | 1.16274 | 2.78206 | ± | 1.71452 | 4.62467 | ± | 1.87599 | 3.03429 | ± | 0.697187 |
| *Il12bv2* | 3.40499 | ± | 1.18354 | 3.01696 | ± | 0.607699 | 3.00656 | ± | 1.22741 | 2.17959 | ± | 1.49474 | 3.50571 | ± | 2.36283 | 3.05674 | ± | 1.78463 |
| *Il15* | 1.39863 | ± | 0.567493 | 1.12423 | ± | 0.62683 | 0.943259 | ± | 0.449717 | 1.43556 | ± | 0.696745 | 1.34973 | ± | 0.844873 | 1.50843 | ± | 0.622431 |
| *Il1b* | 2.96561 | ± | 1.2496 | 3.4158 | ± | 0.795177 | 3.26933 | ± | 0.925525 | 2.16382 | ± | 0.861373 | 3.43169 | ± | 0.975761 | 1.6211 | ± | 1.89376 |
| *Il33* | 1.39571 | ± | 0.703506 | 1.6274 | ± | 0.472728 | 1.75723 | ± | 1.35213 | 0.754966 | ± | 0.52094 | 1.21082 | ± | 0.756005 | 1.42205 | ± | 0.49094 |
| *Il4* | 7.87526 | ± | 1.08795 | 7.8781 | ± | 1.18465 | 7.20291 | ± | 1.91989 | 5.86378 | ± | 2.85096 | 8.64607 | ± | 1.6937 | 7.72869 | ± | 1.82896 |
| *Irf3* | 0.73687 | ± | 0.256585 | 0.227002 | ± | 0.153433 | 0.489527 | ± | 0.393534 | 0.362366 | ± | 0.317549 | 0.72204 | ± | 0.434359 | 0.377128 | ± | 0.238625 |
| *Itgax_v2* | 2.92331 | ± | 1.36558 | 2.6656 | ± | 1.53379 | 3.22376 | ± | 0.389728 | 1.60957 | ± | 1.43889 | 2.96566 | ± | 0.816691 | 1.82879 | ± | 1.20577 |
| *Muc1* | 4.02624 | ± | 0.569321 | 3.13915 | ± | 1.68584 | 4.50006 | ± | 2.19094 | 3.2886 | ± | 0.810549 | 4.5973 | ± | 0.920968 | 4.07019 | ± | 0.621835 |
| *Muc2* | 4.82328 | ± | 0.240429 | 4.35103 | ± | 0.341767 | 3.87123 | ± | 1.73177 | 4.5921 | ± | 0.596624 | 4.69746 | ± | 0.499255 | 5.02333 | ± | 0.601846 |
| *Muc4* | 3.4735 | ± | 0.663908 | 3.19853 | ± | 0.652329 | 3.36119 | ± | 0.395139 | 2.93658 | ± | 0.955347 | 2.53603 | ± | 1.57743 | 3.26048 | ± | 0.671404 |
| *Myd88* | 3.51126 | ± | 0.969172 | 4.14535 | ± | 0.575619 | 3.73196 | ± | 0.636208 | 3.11061 | ± | 1.726 | 4.10218 | ± | 0.627608 | 4.2505 | ± | 0.295649 |
| *Nfkb* | 0.75577 | ± | 0.308903 | 0.62393 | ± | 0.377842 | 0.708528 | ± | 0.450174 | 0.5143 | ± | 0.369616 | 1.14474 | ± | 0.639526 | 0.852823 | ± | 0.266034 |
| *Nfkbia* | 1.338 | ± | 0.326757 | 0.829078 | ± | 0.376109 | 0.922426 | ± | 0.684612 | 0.776574 | ± | 0.383333 | 1.03977 | ± | 0.789422 | 1.02675 | ± | 0.339691 |
| *Nos2* | 7.50845 | ± | 0.902502 | 7.56407 | ± | 1.03047 | 6.81985 | ± | 3.03654 | 6.87716 | ± | 1.31039 | 6.01713 | ± | 1.78676 | 6.5498 | ± | 1.63352 |
| *Ocld* | 0.895668 | ± | 0.304435 | 0.761401 | ± | 0.34452 | 0.629051 | ± | 0.357214 | 0.883744 | ± | 0.335179 | 0.820914 | ± | 0.701712 | 1.04434 | ± | 0.232716 |
| *Pla2g2a* | 1.73799 | ± | 0.248477 | 1.58782 | ± | 0.346513 | 1.30229 | ± | 0.752767 | 1.76545 | ± | 0.786057 | 1.31829 | ± | 0.288205 | 1.75902 | ± | 0.490394 |
| *Ppara* | 0.980108 | ± | 0.4318 | 0.861312 | ± | 0.280125 | 0.630071 | ± | 0.320995 | 0.839632 | ± | 0.638009 | 1.12965 | ± | 0.814316 | 1.41236 | ± | 0.788716 |
| *Pparg* | 0.909564 | ± | 0.127543 | 1.0251 | ± | 0.386908 | 1.1611 | ± | 0.748631 | 0.939392 | ± | 0.327688 | 1.32315 | ± | 0.470393 | 1.08589 | ± | 0.389086 |
| *Pyy* | 1.72562 | ± | 0.329963 | 1.77783 | ± | 0.237112 | 1.44813 | ± | 0.682405 | 1.79034 | ± | 0.179215 | 1.95804 | ± | 0.619559 | 2.14782 | ± | 0.268759 |
| *Reg3a* | 1.34863 | ± | 0.546235 | 1.59964 | ± | 0.719261 | 1.58083 | ± | 0.875591 | 2.60471 | ± | 0.561719 | 2.02637 | ± | 0.758529 | 2.11069 | ± | 0.422495 |
| *Reg3g* | 5.18436 | ± | 1.00916 | 5.25012 | ± | 0.855927 | 4.62287 | ± | 2.60765 | 5.65871 | ± | 1.85246 | 4.11904 | ± | 1.77286 | 4.68744 | ± | 1.57037 |
| *Reg3g_v2* | 4.51148 | ± | 1.01777 | 4.52426 | ± | 0.835215 | 4.35087 | ± | 1.97443 | 5.0078 | ± | 1.58836 | 3.47723 | ± | 1.69007 | 4.45269 | ± | 1.53472 |
| *Retnlb* | 5.78405 | ± | 2.20649 | 6.95624 | ± | 1.40065 | 6.82146 | ± | 2.71185 | 7.53507 | ± | 3.35188 | 5.55506 | ± | 3.44747 | 5.36093 | ± | 3.19285 |
| *Rorc* | 4.41234 | ± | 1.19053 | 4.74842 | ± | 1.57034 | 4.62682 | ± | 1.06184 | 3.22494 | ± | 1.49738 | 3.70074 | ± | 0.932698 | 4.34015 | ± | 0.865792 |
| *Stat4* | 4.0392 | ± | 1.40143 | 3.60135 | ± | 1.03696 | 3.87303 | ± | 1.08897 | 2.29627 | ± | 1.08424 | 3.96776 | ± | 2.59092 | 2.5991 | ± | 2.06357 |
| *Stat5* | 1.43091 | ± | 0.875924 | 1.09693 | ± | 0.63995 | 1.27656 | ± | 0.621225 | 0.974467 | ± | 0.694384 | 1.43269 | ± | 1.52689 | 1.2783 | ± | 0.164742 |
| *Tff3* | 1.02828 | ± | 0.254541 | 0.82916 | ± | 0.261041 | 0.632647 | ± | 0.416006 | 0.96188 | ± | 0.359825 | 1.0568 | ± | 0.587496 | 1.27663 | ± | 0.323435 |
| *Timp1* | 2.35102 | ± | 1.14238 | 2.59386 | ± | 0.942776 | 2.09109 | ± | 0.707875 | 1.9865 | ± | 0.746499 | 2.50504 | ± | 1.66247 | 1.82696 | ± | 1.33302 |
| *Tlr2* | 5.36329 | ± | 0.807493 | 5.03155 | ± | 2.23906 | 5.50507 | ± | 1.53404 | 4.89837 | ± | 1.14548 | 5.14375 | ± | 1.05656 | 4.78757 | ± | 1.32244 |
| *Tlr3* | 1.11297 | ± | 0.314433 | 0.925313 | ± | 0.404238 | 0.991633 | ± | 0.246804 | 0.802615 | ± | 0.395786 | 0.813856 | ± | 0.213922 | 1.08 | ± | 0.160348 |
| *Tlr4* | 2.69297 | ± | 0.440309 | 3.17892 | ± | 0.183288 | 2.5846 | ± | 1.19727 | 2.6517 | ± | 0.428407 | 3.1252 | ± | 0.596489 | 2.66892 | ± | 0.407044 |
| *Tlr5* | 1.2584 | ± | 0.365974 | 1.30831 | ± | 0.502993 | 0.921251 | ± | 0.411076 | 0.996791 | ± | 0.629639 | 1.3433 | ± | 0.807502 | 1.56866 | ± | 0.509626 |
| *Tnfa* | 2.64496 | ± | 0.52079 | 2.40731 | ± | 0.951266 | 2.82671 | ± | 0.66223 | 2.10247 | ± | 1.12938 | 2.85948 | ± | 1.50841 | 1.85436 | ± | 0.855212 |
| *Tnfsf15* | 4.02892 | ± | 0.800428 | 2.64646 | ± | 0.867797 | 2.77399 | ± | 1.42953 | 3.81741 | ± | 1.7141 | 4.091 | ± | 0.635604 | 4.13053 | ± | 1.612 |
| *Zbtb16* | 3.51807 | ± | 1.25423 | 2.96976 | ± | 1.40093 | 2.57883 | ± | 1.22694 | 1.94828 | ± | 1.36196 | 3.56997 | ± | 2.25242 | 3.73877 | ± | 2.02335 |
