# Supplementary material for: The Impact of Human Milk Oligosaccharides on Antibiotic-Induced Microbial Dysbiosis and Gut Inflammation in Mice
Source: Antibiotics (Basel). 2025 May 10;14(5):488. doi: 10.3390/antibiotics14050488 (PMC12108310; doi:10.3390/antibiotics14050488)
Supplement: Supplementary file 1 [file antibiotics-14-00488-s001.zip › Figure S1 Weight.pdf]

Figure S1

(A) Weight curves after weekly weighing of female BALB/cJBomTac mice supplied in their drinking water with HMO's either as 2'FL alone or 2'FL and DFL in combination, either with or without ampicillin for three weeks from the age of four weeks. The control mice received neither HMO or ampicillin. Areas under curves for the periods Day 0-7, day 0-14 and day 0-22 were tested by a three-way ANOVA (B) and post hoc Tukey's comparisons (C). p values were considered significant if $p < 0.05$ (bold) and borderline if $p < 0.10$ (italics).

A

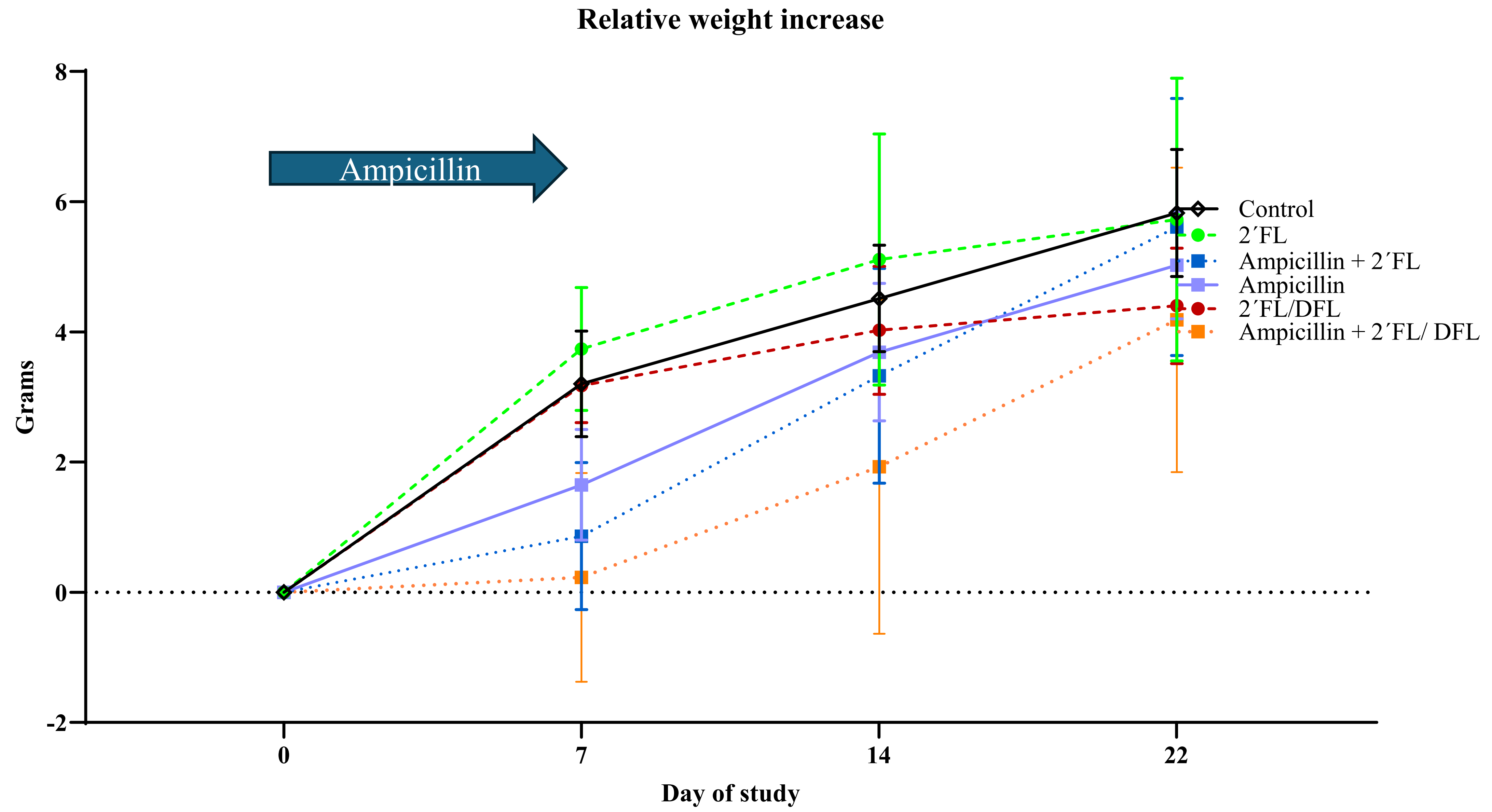

B

	Day 0-7	Day 0-14	Day 0-22
		Three-way ANOVA	
Ampicillin	0.000	0.001	0.001
2'FL (alone)	n.s.	n.s.	n.s.
2'FL + DFL	*0.068*	*0.068*	*0.089*

Tukey' posthoc comparisons

C

	2'FL	2'FL + DFL	Ampicillin	Ampicillin + 2'FL	Ampicillin + 2'FL + DFL	2'FL	2'FL + DFL	Ampicillin	Ampicillin + 2'FL	Ampicillin + 2'FL + DFL	2'FL	2'FL + DFL	Ampicillin	Ampicillin + 2'FL
Control	n.s.	n.s.	0.043	0.001	0.000	n.s.	n.s.	n.s.	n.s.	0.044	n.s.	n.s.	n.s.	n.s.
2'FL		n.s.	0.002	0.000	0.000		n.s.	n.s.	n.s.	0.009		n.s.	n.s.	n.s.
2'FL + DFL			0.049	0.000	0.001			n.s.	n.s.	n.s.			n.s.	n.s.
Ampicillin				n.s.	*0.097*				n.s.	n.s.				n.s.
Ampicillin + 2'FL					n.s.					n.s.				
