# Supplementary material for: The Impact of Human Milk Oligosaccharides on Antibiotic-Induced Microbial Dysbiosis and Gut Inflammation in Mice
Source: Antibiotics (Basel). 2025 May 10;14(5):488. doi: 10.3390/antibiotics14050488 (PMC12108310; doi:10.3390/antibiotics14050488)

Figure S2

Water intake per cage in female BALB/cJBomTac mice supplied with drinking water HMO's either as 2'FL alone or combined 2'FL and DFL, with or without ampicillin for three weeks from the age of four weeks. The control mice received neither HMO or ampicillin. Areas under curves were tested by a three-way ANOVA. p values were considered significant if $p < 0.05$ (bold).

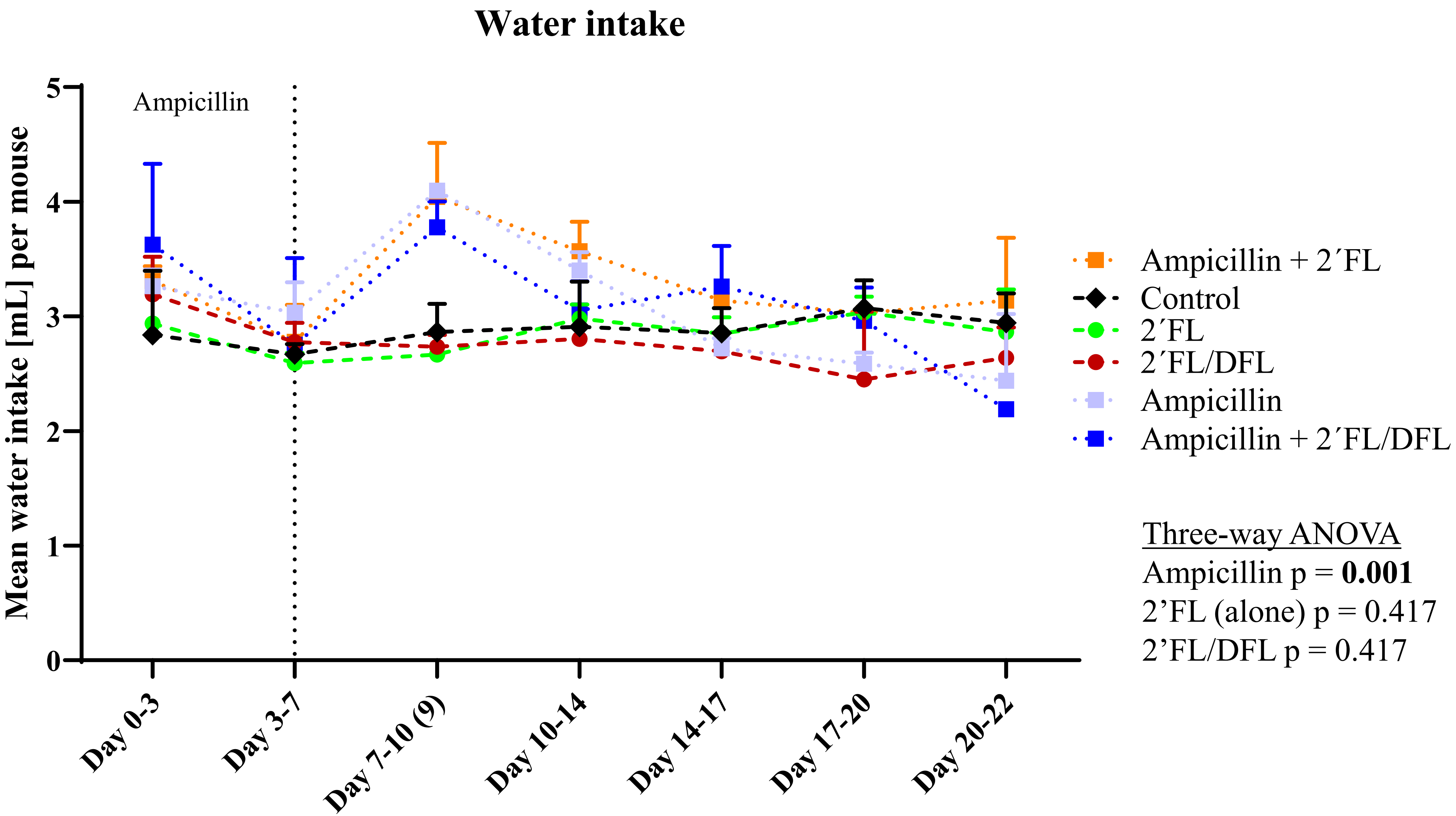

Supplement: Supplementary file 1 [file antibiotics-14-00488-s001.zip › Figure S2 Water intake.pdf]
