# Supplementary material for: The Impact of Human Milk Oligosaccharides on Antibiotic-Induced Microbial Dysbiosis and Gut Inflammation in Mice
Source: Antibiotics (Basel). 2025 May 10;14(5):488. doi: 10.3390/antibiotics14050488 (PMC12108310; doi:10.3390/antibiotics14050488)
Supplement: Supplementary file 1 [file antibiotics-14-00488-s001.zip › Table S1 Over all family abundances.docx]

Table S1

Bacterial families identified by 16S amplicon sequencing feces of 48 female BALB/cJBomTac mice divided into six groups of eight mice with four mice in each of two cages supplied in their drinking (tap) water with HMO’s either as 2’FL alone or 2’FL and DFL in combination, either with or without ampicillin for three weeks from the age of four weeks. Abundances over all from all time periods (Table S2) or from different time points (Table 1) are interpreted as significant increase (red) or decrease (blue). Families significantly impacted by either ampicillin, the HMOs or both are grey scaled.

|  | No ampicillin | | | Ampicillin | | | q-values | | | |
| --- | --- | --- | --- | --- | --- | --- | --- | --- | --- | --- |
|  | **Control** | **2'FL** | **2'FL + DFL** | **Ampicillin** | **+.2'FL** | **+.2'FL/DFL** | **Day** | **Ampicillin** | **2'FL** | **2'FL/DFL** |
| Actinobacteria;Coriobacteriia;Coriobacteriales;Coriobacteriaceae | 0.0012607 | 0.0016344 | 0.0012141 | 0.000654 | 0.0004382 | 0.0010931 | **0.001** | **0.017** | 0.952 | 0.520 |
| Bacteroidetes;Bacteroidia;Bacteroidales; | 0.0005996 | 0.0007598 | 0.0004448 | 0.0001263 | 0.0000474 | 0.0000556 | 0.113 | **0.000** | 1.000 | 0.170 |
| Bacteroidetes;Bacteroidia;Bacteroidales;AC16063Unknown | 0.0183178 | 0.0157173 | 0.0168448 | 0.008301 | 0.0031992 | 0.0041914 | 0.000 | **0.009** | 0.530 | 0.794 |
| Bacteroidetes;Bacteroidia;Bacteroidales;Bacteroidaceae | 0.0270309 | 0.0236923 | 0.0167464 | 0.0545865 | 0.129919 | 0.141577 | 0.001 | 0.402 | 0.103 | 0.872 |
| Bacteroidetes;Bacteroidia;Bacteroidales;Muribaculaceae | 0.344592 | 0.41119 | 0.494248 | 0.302942 | 0.223805 | 0.292203 | 0.000 | **0.033** | 0.165 | **0.000** |
| Bacteroidetes;Bacteroidia;Bacteroidales;Odoribacteraceae | 0.0059102 | 0.007224 | 0.0046885 | 0.001751 | 0.0007488 | 0.0009172 | 0.120 | **0.000** | 0.932 | 0.170 |
| Bacteroidetes;Bacteroidia;Bacteroidales;Prevotellaceae | 0.0526133 | 0.0435521 | 0.0278412 | 0.015196 | 0.0221635 | 0.006122 | *0.093* | **0.000** | 0.567 | **0.020** |
| Bacteroidetes;Bacteroidia;Bacteroidales;Rikenellaceae | 0.0990021 | 0.0907712 | 0.0832253 | 0.03355 | 0.0224472 | 0.0636811 | *0.053* | **0.000** | 0.866 | 0.398 |
| Cyanobacteria;VAmpicillinirovibrio;FR888536;FR888536 | 0.0008432 | 0.0004249 | 0.0003281 | 0.0004647 | 0.0002642 | 0.0001118 | **0.000** | 0.472 | 0.700 | 0.140 |
| Firmicutes;Bacilli;Lactobacillales;Lactobacillaceae | 0.040842 | 0.0458302 | 0.109728 | 0.0094027 | 0.0149132 | 0.0224433 | **0.021** | **0.000** | 0.530 | **0.000** |
| Firmicutes;Bacilli;Lactobacillales;Streptococcaceae | 0.0003418 | 0.0003521 | 0.0002487 | 0.0002613 | 0.0001326 | 0.0002129 | **0.000** | 0.384 | 0.567 | 0.566 |
| Firmicutes;Clostridia;Clostridiales; | 0.0001341 | 0.0001445 | 0.0001626 | 0.000134 | 0.0004261 | 0.0001414 | **0.038** | 0.587 | 0.519 | 0.369 |
| Firmicutes;Clostridia;Clostridiales;Christensenellaceae | 0.005245 | 0.005968 | 0.0042986 | 0.0022128 | 0.0018756 | 0.0015023 | 0.255 | **0.004** | 1.000 | 0.302 |
| Firmicutes;Clostridia;Clostridiales;Dehalobacterium | 0.0002407 | 0.0002547 | 0.0001424 | 0.0000873 | 0.0000693 | 0.000057 | 0.339 | **0.025** | 0.952 | 0.217 |
| Firmicutes;Clostridia;Clostridiales;Lachnospiraceae | 0.28141 | 0.236344 | 0.155045 | 0.355406 | 0.265739 | 0.1783 | **0.000** | **0.033** | 0.258 | **0.000** |
| Firmicutes;Clostridia;Clostridiales;Mogibacterium | 0.0001599 | 0.0001766 | 0.0000785 | 0.0000768 | 0.0000685 | 0.0000517 | **0.000** | **0.011** | 0.801 | **0.022** |
| Firmicutes;Clostridia;Clostridiales;Peptococcaceae | 0.0002875 | 0.0001631 | 0.0000449 | 0.0001969 | 0.0000605 | 0.0000747 | **0.000** | 0.751 | 0.530 | **0.030** |
| Firmicutes;Clostridia;Clostridiales;Ruminococcaceae | 0.0727792 | 0.0725453 | 0.0437353 | 0.0722534 | 0.0472523 | 0.0435955 | **0.000** | 0.800 | 0.502 | **0.020** |
| Firmicutes;Erysipelotrichi;Erysipelotrichales;Erysipelotrichaceae | 0.0002204 | 0.0002367 | 0.0002285 | 0.001161 | 0.0072908 | 0.0022539 | **0.001** | 0.797 | *0.069* | 0.170 |
| Proteobacteria;Alphaproteobacteria;; | 0.0004164 | 0.0002191 | 0.0001658 | 0.0037144 | 0.007923 | 0.0040691 | **0.000** | *0.056* | 0.530 | 0.298 |
| Proteobacteria;Alphaproteobacteria;Rhodospirillales;Rhodospirillaceae | 0.000462 | 0.0008652 | 0.0008893 | 0.0000599 | 0.0000938 | 0.0001115 | 0.541 | **0.009** | 0.997 | 0.407 |
| Proteobacteria;Betaproteobacteria;Burkholderiales;Comamonadaceae | 0.0000009 | 0 | 0 | 0 | 0 | 0.0044282 | **0.007** | 0.453 | 0.567 | 0.170 |
| Proteobacteria;Deltaproteobacteria;Desulfovibrionales;Desulfovibrionaceae | 0.0005698 | 0.0004773 | 0.0003765 | 0.0001472 | 0.0000947 | 0.0001239 | 0.120 | **0.000** | 0.997 | 0.369 |
| Proteobacteria;Gammaproteobacteria;Enterobacterales;Enterobacteriaceae | 0.0001773 | 0.000132 | 0.0002377 | 0.0004399 | 0.0663539 | 0.0227907 | **0.000** | 0.587 | **0.034** | 0.182 |
| Proteobacteria;Gammaproteobacteria;Pseudomonadales;Pseudomonadaceae | 0 | 0.0000019 | 0.0000021 | 0.0000015 | 0.0000046 | 0.0860774 | **0.000** | 0.384 | 0.530 | **0.000** |
| Saccharibacteria_TM7;Saccharimonas;Saccharimonas;Saccharimonas | 0.000231 | 0.0004415 | 0.0006124 | 0.0001324 | 0.0001269 | 0.000051 | 0.428 | **0.033** | 0.567 | 0.170 |
| Proteobacteria;Betaproteobacteria;Burkholderiales;Comamonadaceae | 0.0000009 | 0 | 0 | 0 | 0 | 0.0044282 | **0.007** | 0.453 | 0.567 | 0.170 |
| Proteobacteria;Deltaproteobacteria;Desulfovibrionales;Desulfovibrionaceae | 0.0005698 | 0.0004773 | 0.0003765 | 0.0001472 | 0.0000947 | 0.0001239 | 0.120 | **0.000** | 0.997 | 0.369 |
| Proteobacteria;Gammaproteobacteria;Enterobacterales;Enterobacteriaceae | 0.0001773 | 0.000132 | 0.0002377 | 0.0004399 | 0.0663539 | 0.0227907 | **0.000** | 0.587 | **0.034** | 0.182 |
| Proteobacteria;Gammaproteobacteria;Pseudomonadales;Pseudomonadaceae | 0 | 0.0000019 | 0.0000021 | 0.0000015 | 0.0000046 | 0.0860774 | **0.000** | 0.384 | 0.530 | **0.000** |
| Saccharibacteria_TM7;Saccharimonas;Saccharimonas;Saccharimonas | 0.000231 | 0.0004415 | 0.0006124 | 0.0001324 | 0.0001269 | 0.000051 | 0.428 | **0.033** | 0.567 | 0.170 |
